# How Do People Generalize Causal Relations over Objects? A Non-parametric Bayesian Account

**DOI:** 10.1007/s42113-021-00124-z

**Published:** 2021-11-30

**Authors:** Bonan Zhao, Christopher G. Lucas, Neil R. Bramley

**Affiliations:** 1 Department of Psychology, The University of Edinburgh, South Bridge, Edinburgh, EH8 9YL UK; 2 School of Informatics, The University of Edinburgh, Edinburgh, UK

**Keywords:** Causal reasoning, Generalization, Bayesian models, Inductive bias, Program induction, Dirichlet process

## Abstract

How do people decide how general a causal relationship is, in terms of the entities or situations it applies to? What features do people use to decide whether a new situation is governed by a new causal law or an old one? How can people make these difficult judgments in a fast, efficient way? We address these questions in two experiments that ask participants to generalize from one (Experiment 1) or several (Experiment 2) causal interactions between pairs of objects. In each case, participants see an agent object act on a recipient object, causing some changes to the recipient. In line with the human capacity for few-shot concept learning, we find systematic patterns of causal generalizations favoring simpler causal laws that extend over categories of similar objects. In Experiment 1, we find that participants’ inferences are shaped by the order of the generalization questions they are asked. In both experiments, we find an asymmetry in the formation of causal categories: participants preferentially identify causal laws with features of the agent objects rather than recipients. To explain this, we develop a computational model that combines program induction (about the hidden causal laws) with non-parametric category inference (about their domains of influence). We demonstrate that our modeling approach can both explain the order effect in Experiment 1 and the causal asymmetry, and outperforms a naïve Bayesian account while providing a computationally plausible mechanism for real-world causal generalization.

## Introduction

People readily generalize from familiar causal relationships to novel ones, using the features of prospective objects as a guide. For example, if you need to pound a nail but cannot find a hammer, you might pick up a nearby brick instead, reasoning that it will “do the job”; a child who has recently discovered drawing with colored chalks on paper may then explore the extent of this new power, using them to draw on the walls, the mirror, or even her bed sheets. These kinds of everyday actions call upon what we call *object-based causal generalization*.

Indeed, a fundamental goal of cognition is to generalize from limited experience so as to behave appropriately in unpredictable future tasks and situations. We achieve this, in part, by constructing models of the environment that provide reliable predictions (Craik, 1952; Hume, 1740). While a wealth of research has been devoted to studying how children and adults acquire causal beliefs (e.g., Bramley et al., 2015; Gopnik et al., 2007; Griffiths & Tenenbaum 2009; Kemp et al., 2012; Sloman 2005), understand the world using core knowledge of objects (Baillargeon, 1995; Spelke, 1990; Spelke & Kinzler, 2007), and generalize functional properties (e.g., Goodman et al.^2008^; Lucas et al., 2015; Shepard 1987; Tenenbaum & Griffiths 2001; Wu et al., 2018), the interplay between causality, object concepts and generalization has received less attention. On the face of it, this is surprising. If causal beliefs did not frequently extend to novel entities and situations, they would be of limited use to us. Therefore, a key aspect of successful causal learning is to generalize causal relations appropriately to new situations that are related but nonidentical to past experiences. Generalization, on the other hand, could not be successful without tapping into what Sloman calls Nature’s “invariants” (Sloman, 2005), the true causal laws that govern both experienced and novel situations. While research has explored the interplay between causality and generalization using hierarchical Bayesian models (e.g., Kemp et al., 2010; Goodman et al.^2011^; Griffiths & Tenenbaum 2009), this computational level approach (Marr, 1982) is limited in its ability to capture psychological processes due to its inherent intractability (Kwisthout & Van Rooij, 2020; Van Rooij, 2008).

**Fig. 1:**
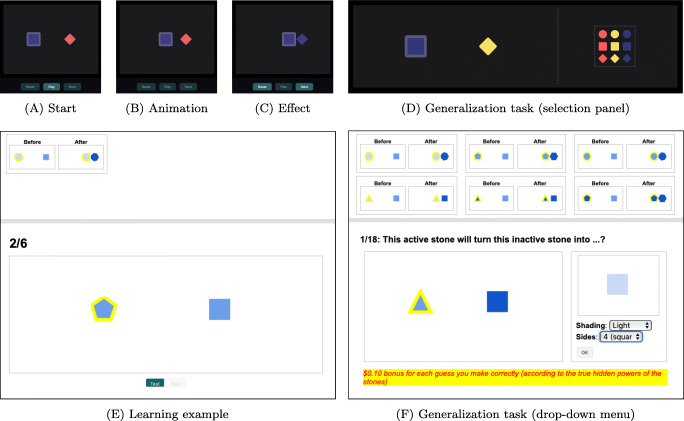
Our object-based causal generalization task interfaces. A–D: Experiment 1. A–C step through an example learning scene animation, and D shows a generalization task consisting of novel objects (left) and a selection panel (right), in which learners select from a set of possible predictions about the appearance of the recipient after the causal interaction. E–F: Experiment 2. Summaries of previous learning examples are shown at the top of the screen. E shows one animated effect similar to A–C. In F, generalization predictions are elicited by selecting from two drop-down menus (one per feature)

In this paper, we explore how people generalize causal relations from observed interactions between pairs of simple geometric objects via a “magic stone” task. In it, participants test causal relationships between a magic stone (the agent) and a normal stone (the recipient) by watching the agent object moves toward the recipient object, and upon touching each other the recipient object changes into a result form (Fig. 1A–C).^1^ Participants are asked to make predictions about new pairs of objects “This new magic stone will turn this new normal stone into ...?” (Fig. 1D). Observing objects interacting naturally invokes causal perceptions. For instance, Michotte (1963) discovered the “launching” phenomenon, in which participants directly perceive a causal influence connecting two objects that act in sequential order: If object A moves toward a stationary object B, and if around when A touches B, A stops moving and B starts to move, participants report that they see object A cause object B to move (see also Gordon et al., 1990; Leslie and Keeble 1987; Scholl & Tremoulet^2000^). Similarly, the animated agent-recipient setup in our task lays out an overtly causal framing, allowing us to probe the inductive biases and cognitive processes that are distinctive to causal reasoning. Unlike previous work in causal induction (e.g., Griffiths & Tenenbaum 2009), this abstract setting minimizes the influence of specific domain priors and background knowledge. Our experimental framework can be viewed as a conceptual extension to classic Blicket experiments in developmental psychology (e.g., Gopnik & Sobel 2000; Kemp et al., 2010; Lucas & Griffiths 2010), and we discuss this connection in detail in the “General Discussion” section.

With these causal and object-based representations of the task, we open up a large space of scenarios and possibilities that demand sophisticated combinatorial reasoning, especially in terms of generalization. The relevant inference here is not about whether the agent object is the cause of the recipient object’s change or not (e.g., Cheng^1997^; Jenkins & Ward 1965; Pearl 2000; Sloman & Lagnado 2005; Tenenbaum & Griffiths 2000); instead, we are interested in a different question: Given an observation where a particular agent object causes a particular change in a particular recipient object, how do people generalize this causal interaction to novel objects, where both agent and recipient may share more or fewer features with those in the original observation? One might conjecture that “square agents cause recipients to turn blue,” which applies to all the square agents but says nothing about circles; or a more general causal principle, perhaps “all agents cause recipients to take their color,” which can apply universally to all the potential objects. While the structure of such rules is logical, the domain framing brings causal inductive biases to bear. For instance, we can probe the possibility that people view agents’ features as playing different functional roles to recipients’ features in causal interactions.

In the following sections, we introduce a computational modeling framework for object-based causal inference in the spirit of Griffiths and Tenenbaum (2009) and Lucas and Griffiths (2010), but with a more expressive hypothesis space that better captures the diverse inferences people can make. It draws on non-parametric approaches to category and function learning to account for similarity-based generalization predictions. The normative version, called LoCaLa (Local Causal Laws), compares each generalization trial against all the learning examples in order to assign causal categories to new observations. A “resource-rational” (Goodman et al., 2008; Sanborn et al., 2010) variant of it, the LoCaLaPro (Local Causal Laws Process) model, shares causal categories among generalization trials, and only updates a causal category when it cannot explain a novel observation. After introducing these models, we report on two experiments that shed light on previously unexplored inductive biases in causal learning, and allow us to evaluate our models and the ideas that motivate them. We find that our local laws and particularly our new process model better explain our behavioral data than a purely normative account, including explaining a novel generalization-order effect observed in Experiment 1. We conclude with a discussion of our model’s scope and limitations and highlight some potential future directions.

## Computational Modeling Framework

**Fig. 2:**
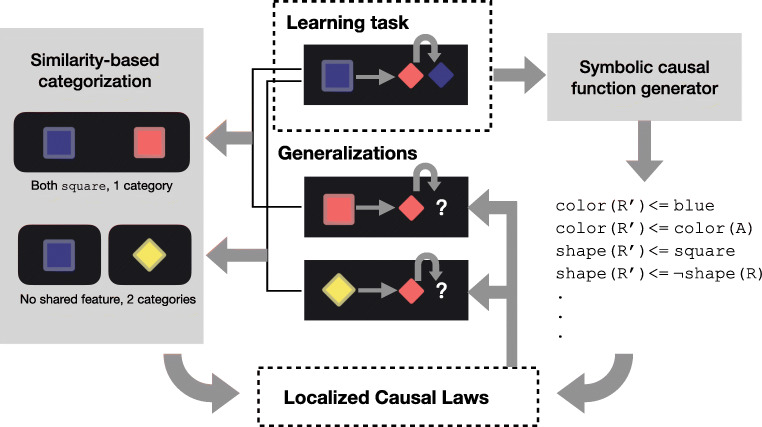
To model how people make object-based causal generalization predictions (middle), we combine program induction about the hidden causal laws (right) with non-parametric category inference about their domains of influence (left). Together, they form latent causal categories that guide generalization predictions (dark arrows from bottom to middle)

Causal generalization involves two forms of induction: (1) Inferring what causal relationship is at work in an observed setting, known as causal learning or causal induction, and (2) Inferring the domain to which a causal law applies, closely related to categorization. In correspondence, our computational modeling framework integrates a generative grammar to model the vast space of possible causal relationships (causal laws), and Bayesian non-parameteric categorization process that accounts for the domain of influence for those causal relationships (Fig. 2). Together, they provide a principled account for causal generalization over novel interacting objects.

### Causal Laws

To a first approximation, objects are identifiable by their features and causal powers (Aristotle, 322/1998; Gopnik et al., 2004). Adults find basic features of objects, such as color, shape, and orientation to be salient cues for information selection (Treisman & Gelade, 1980; Treisman & Paterson, 1984). Therefore, we represent objects in terms of their observable features, and model interactions between objects using causal functions. For example, we can read an object’s color by color(*o*) is red. When an agent acts on a recipient and causes the recipient to change, we model this with a causal function *f* that takes the agent (*a*) and recipient’s initial state (*r*) as input, and outputs the final state of the recipient ($r^{\prime }$), which we call the *result*. Depending on the situation, real causal interactions could result in changes to the form of the agent object as well. However, given the examples in Fig. 1, we restrict our focus to *f*(*a*,*r*) ⇒ *r*^′^ in this paper. Naturally, a causal function defines the result *r*^′^ by specifying its features, potentially conditional on specific features of *a* and *r*. Take an everyday understanding of paint for an example: When applied to a wall, paint causes that wall to take the color of the paint. We can formalize this as a function $f(\texttt {paint}, \texttt {wall}) \Rightarrow \texttt {wall}^{\prime }$, where color(wall^′^) ⇐color(paint). Note that arrow ⇒ reads as “gives” or “produces”: *f*(*a*,*r*) ⇒ *r*^′^ says that function *f*(*a*,*r*) produces result object *r*^′^. Arrow ⇐ is an assignment operation: color(wall^′^) ⇐color(paint) means that color of the paint is assigned to (color of) the wall.

Griffiths and Tenenbaum (2009) proposed a computational level, hierarchical Bayesian model (HBM) framework for causal inference where structured domain knowledge restricts the space of possible or plausible causal relationships. However, this work focused on statistical relationships between variables rather than interactions between objects. Furthermore, the space of possible causal functions in natural settings is clearly intractable, posing a serious computational challenge for any bounded learner. Therefore, more recent accounts of causal learning have treated causal inference as practically constituting a search problem in a large multi-modal theory space (Bramley et al., 2017), and utilize generative grammars and program induction ideas to cover the open ended space of a learner’s possible theories and hypotheses (Goodman et al., 2008, 2011; Bramley et al., 2021), as well as the human preference for simpler causal explanations (cf. Feldman 2000).

Following this approach, we use a Probabilistic Context-Free Grammar (PCFG; Ginsburg 1966) to define a prior over possible causal relationships (causal laws, right column in Fig. 2). A PCFG is defined by a tuple (Γ,Θ,*T*), where Γ is a set of production rules, Θ a set of production probabilities, and *T* a set of transition symbols. Our example grammar $\mathcal {G}$ (Table 1) has a set of transition symbols *T* = {*S*,*A*,*B*,*C*,*D*,*E*}, where *S* is the “Start” symbol by convention. Starting from symbol *S*, grammar $\mathcal {G}$ follows the production rules to generate expressions, and stops when there are no transition symbols anymore in the expression. Production rules Γ define how transition symbols transform. Production probabilities Θ assign a probability distribution for each transition symbol’s possible transformations. For simplicity, we assume uniform production probabilities: let Γ_*L*_ be the set of all production rules that start with symbol *L* ∈ *T* (i.e., production rules in the form of *L* → *K*, where *K* can be any symbol in grammar $\mathcal {G}$), the transition probability for each *l* ∈Γ_*L*_ is simply $\frac {1}{|{\Gamma }_{L}|}$. For example, on the “Reference” row in Table 1, symbol *C* can either follow production rule *C* → *D* and produce *D*, or follow production rule *C* → *E* and produce *E*. We thus assume symbol *C* has 0.5 probability to become *D*, and 0.5 probability to become *E*.

**Table 1:** Example probabilistic grammar $\mathcal {G}$

Production rules				Example generation
Start				*S*
Bind feature	$S \to \lambda _{\phi _{i}}:A, {\Phi }$			*λ*_color_ : *A*
Bind additional	*A* → *B*	∣	*A* →and(*B*,*S*)	*λ*_color_ : *B*
Relation	*B* → *ϕ*_*i*_(*r*^′^) ⇐ *C*	∣	*B* → *ϕ*_*i*_(*r*^′^) ⇐¬*C*	*λ*_color_ : color(*r*^′^) ⇐¬*C*
Reference	*C* → *D*	∣	*C* → *E*	*λ*_color_ : color(*r*^′^) ⇐¬*D*
Relative reference	*D* → *ϕ*_*i*_(*a*)	∣	*D* → *ϕ*_*i*_(*r*)	color(*r*^′^) ⇐¬color(*r*)
Absolute reference	$E \to \texttt {value}^{\phi _{i}}$			

Note: *ϕ*_*i*_ denotes the *i* th feature in the set of all observable object features Φ. The lambda abstraction in the “bind feature” production rule samples a feature without replacement from the set of all features, and binds this feature *ϕ*_*i*_ to the rest of the generation: *ϕ*_*i*_ in *D* uses the same feature selected in *A*, and value in *E* is sampled uniformly from the support of feature *ϕ*_*i*_. Production probabilities are omitted from the table because we assume a uniform prior.

We now walk through an example for our grammar $\mathcal {G}$ in Table 1. Starting from symbol *S*, production rule $S \to \lambda _{\phi _{i}}:A, {\Phi }$ samples a feature uniformly from the set of all observable features (in the task) and binds it to the production. Let’s assume we sampled feature color with probability 0.5 (out of Φ = {color,shape}), and now the expression becomes *λ*_color_ : *A*. Symbol *A* leads two productions: either becomes *B*, or and(*B*,*S*), with uniform prior probability. Assume that with probability 0.5 we retrieve expression *B*. Proceeding to row “Relation,” with probability 0.5 we could arrive at color(*r*^′^) ⇐¬*C*. Then on row “Reference” with 0.5 probability we could get color(*r*^′^) ⇐¬*D*. Finally, symbol *D* produces either color(*a*) or color(*r*) equally likely, and with probability 0.5 we end up with color(*r*^′^) ⇐¬color(*r*): result object’s color is assigned to a color that is different from the recipient’s, i.e., result object changes its color. In total, the probability of producing color(*r*^′^) ⇐¬color(*r*) is 0.5^5^ = 0.03. If at the step of “Reference” we followed production rule *C* → *E* instead, then with probability 0.33 we might sample a color blue (out of value^color^ = {red,yellow,blue}), and the probability of producing color(*r*^′^) ⇐¬blue is 0.5^4^ × 0.33 = 0.02.

It is worth noting that by design, this grammar is inherently more likely to produce simpler expressions. For the “Bind additional” rule *A* →and(*B*,*S*) is called with probability 0.5, and thus the number of conjunctions in the final expression follows a geometric decay with only 50% combining more than one assertion, 25% containing more than two, and so on.

Formally speaking, the prior for a given expression is the product of all the productions that produced it:

1 $$ P_{\mathcal{G}}(f) = \prod\limits_{l \in {\Gamma}}(\theta_{l})^{c_{l}} $$

where *𝜃*_*l*_ ∈Θ is the production probability for production rule *l* ∈Γ, and *c*_*l*_ is how many times rule *l* was used for generating causal function *f*.

Grammar $\mathcal {G}$ assigns a prior over a potentially infinite set of causal functions. A causal function defines the result object(s) by describing the result object’s feature values, given the particular agent and recipient object inputs. Take and(color(*r*^′^) ⇐color(*a*),shape(*r*^′^) ⇐square) for example. For an agent *a* that is a red-circle and a recipient *r* that is a blue-pentagon, *r* will become *r*^′^: a red-square. When a causal function *f* involves a negation, it could have produced more than one outcome. For instance, consider a causal function shape(*r*^′^) ⇐¬triangle, any object that is not triangular (and share the same color as *r*) is a possible option for being *r*^′^. We further assume for simplicity that the different potential outcomes are equally probable, and thus likelihood of a data point *d* = (*a*,*r*,*r*^′^) generated by a causal function *f* is given by

2 $$ P(d|f) = P(r'|f, a, r) = \begin{cases} \frac{1}{D(f(a,r))} &\text{if }r' \in D(f(a,r)),\\ 0 &\text{otherwise} \end{cases} $$

where *D*(*f*(*a*,*r*)) refers to the set of all possible result objects coming out of *f* given agent *a* and recipient *r* (*D* stands for domain). We initially assume a likelihood to 0 for any observation (*a*,*r*,*r*^′^)∉*D*(*f*(*a*,*r*)), but later consider “soft” variants in which functional relationships are somewhat fallible.

This framework naturally favors deterministic causal functions that are consistent with the evidence: if a causal function predicts a specific result, when that outcome is indeed observed, likelihood will be 1. In contrast, a causal function that predicts a range of outcomes will inevitably assign a lower likelihood to any one of these. For example, if you observe a recipient turning blue, this is more consistent with a function where the agent invariably turns the recipient blue than with one where the agent turns the recipient to either red or blue. We note that while many of these choices are somewhat arbitrary, or are made for computational convenience with respect to the current task context, the approach itself is highly general and flexible, compatible with many other more or less expressive grammars and production processes embodying stronger or weaker priors.

According to Bayes Theorem, upon seeing some learning data *d*, the posterior distribution over causal functions is

3 $$ P(f|d, \mathcal{G}) \propto P(d|f)P_{\mathcal{G}}(f) $$

If causal functions apply universally to all the objects, Eq. 3 solves the learning and generalization problems at the same time: after updating the prior of causal functions with learning data, the posterior predictive gives generalization predictions for every novel pair of objects. For instance, the animation example in Fig. 1A–C results in a posterior over causal functions favoring color(*r*^′^) ⇐color(*a*), color(*r*^′^) ⇐blue and some other possibilities (recall the set is potentially infinite). Then, in the generalization prediction phase as in Fig. 1D, marginalizing over that posterior leads to a prediction favoring blue-diamond. Formally, upon observing a partial data point *d*^∗^ = (*a*^∗^,*r*^∗^,⋅), an optimal decision can be made by marginalizing over the posterior predictive distribution of each possible *r*^′^^∗^ value:

4 $$ P(r'|d^{*}) = \int P(r'|a^{*}, r^{*}, f)P(f|d,\mathcal{G}) df $$

Grammar $\mathcal {G}$ and Eqs. 1–4 together define our first normative model Universal Causal Laws (UnCaLa).

### Featural Similarity-Based Categorization

However, while it has been argued that we think of causal relationships as “invariant” (Sloman, 2005), in the sense that they apply across contexts and over time, our causal beliefs are so entangled with our concepts and categories that we think of certain kinds of objects as having particular causal powers, and others as being susceptible to particular causal influences. For instance, we may well understand that a bucket of paint can cause almost any surface to take on the agents’ color, but other classes of agents, like jumpers and cucumbers, do not make recipients take their color. Category knowledge thus seems integral to real-world causal inference. If novel encounters involve objects of familiar categories, one can generalize the causal functional relationships and predict likely effects. When objects fall into different categories, however, those causal laws that one category has are not necessarily possessed by the other category.

In fact, while people refer to causal relationships when categorizing objects (Gopnik & Sobel, 2000; Rehder & Hastie, 2001; Rehder, 2003), they also spontaneously use featural and relational information for categorization when no causal information is available (Anderson, 1991; Love et al., 2004; Kemp & Tenenbaum, 2008), and then make causal predictions based on these categories (Kemp et al., 2010), suggesting the widespread assumption that features reflect hidden causal powers.

But what determines the scope of our beliefs about particular causal functional relationships? Intuitively, this could be about the nature of the agent, the recipient, or both. For example a function might apply to a specific agent acting on a broad range of recipients—e.g., water will make most of its potential recipients wet. Conversely, we might conceive of causal relationships that hold for a broad range of agents acting on a specific recipient—e.g., that most things will break an extremely fragile bottle. A relationship could also be both agent-recipient-specific—e.g., an electrical plug will only connect successfully to a socket of matching shape. One dimension of our experiments in this paper is to explore people’s intuitive causal categorization assumptions. To foreshadow, we find a causal asymmetry bias in which agents play the dominant role in determining effects, consistent with White (2006).

We formalize the idea that the pairs of objects may fall into different categories with respect to featural similarities and their roles in the interaction with a Dirichlet Process (DP). We treat one such category as a distribution over objects, and DP defines a prior over a potentially infinitely many categories. Let **d** denote a set of observations, **z** denote a particular set of category memberships, and **w** denote some categorization parameters (weights). We use superscript (*i*) for the *i* th observation: *d*^(*i*)^ for the *i* th data point, *z*^(*i*)^ the causal category assigned to the *i* th observation, *a*^(*i*)^ the agent in the *i* th data point, similarly for *r*^(*i*)^,*r*^′^^(*i*)^, and $\mathbf {w}_{z^{(i)}}$ for the weights associated with category *z*^(*i*)^. Inference about the *i* th observation’s category is given by

5 $$ P(z^{(i)}|\mathbf{d}, \mathbf{w}) \propto P(z_{i}|z^{(-i)})P(a^{(i)}, r^{(i)}|\mathbf{w}_{z^{(i)}}). $$

Equation 5 consists of two parts: *P*(*z*^(*i*)^|*z*^(−*i*)^) reflects our prior expectations about how categories are distributed, and $P(a^{(i)}, r^{(i)}|\mathbf {w}_{z^{(i)}})$ encodes our beliefs about object features and category membership.

In DP, the prior expectation of categories is given by a Chinese Restaurant Process (CRP), controlled by a concentration parameter *α*. A CRP is a stochastic process widely used for creating partitions among entities (Aldous, 1985). It draws on an analogy of sequentially seating infinite incoming customers to infinitely many tables in a Chinese restaurant, where each table is also of infinite capacity. The first observation *d*^(1)^ is always assigned the first category *z*^(1)^; when *i* > 1, the probability for assigning category *z*^(*i*)^ is given by

6 $$  P(z^{(i)} = x|z^{(-i)}) = \begin{cases} \frac{\alpha}{i-1+\alpha}  &\text{if } x \text{ is a new category} \\ \frac{|z^{(j)}|}{i-1+\alpha} &\text{if } x = z^{(j)} \end{cases} $$

where *z*^(*j*)^ is an existing category, and |*z*^(*j*)^| is the number of assigned objects in category *z*^(*j*)^. Parameter *α* is known as the concentration, or dispersion parameter—the larger *α* is, the more likely a new object falls into a new category. Holding the same *α*, categories with more members are preferred as they seem to be more “common.”

Preference for feature similarities can be modeled by a multinomial distribution over feature values. Let [*μ*_1_,…,*μ*_*n*_] be the mean feature vector of a given category where each subscript *k* is a feature value, probability of an object assigned to a particular category according to feature similarities is given by

7 $$ P(o^{(i)}|\theta_{z^{i}}) = \prod\limits_{k=1}^{n} \text{Bernoulli}(o^{(i)}; \mu_{k}) $$

To compute *μ*_*k*_, let $o_{v}=[o_{v_{1}}, \ldots , o_{v_{n}}]$ be the feature values of an object *o*, where each *v* represents a feature value, $o_{v_{i}}=1$ if object *o* has this feature value and $o_{v_{i}}=0$ otherwise. For a category *z* = {*o*^(*i*)^,…,*o*^(*m*)^}, $z_{v} := {\sum }_{j=1}^{m} o_{v}^{(j)}$, which can be written as $z_{v}= [z_{v_{1}}, \ldots , z_{v_{n}}]$, where $z_{v_{i}} = {\sum }_{j=1}^{m} o^{(j)}_{v_{i}}$. Mean feature $\mu _{k} := \frac {z_{v_{k}}}{{\sum }_{l=1}^{n}(z_{v_{l}})}$. We assign a Dirichlet prior to this multinomial distribution in order to capture how important feature similarity is in forming categories. Without leaning toward any specific feature, the prior distribution over mean features is simply Dir(*β*).

It is not obvious whether mean features should be drawn from the agent object, recipient object, or both, therefore we introduce one more hyperparameter *γ*, referring to the probability that mean feature is purely based on the agent: when *γ* = 1, categorization is only grounded on the agent objects, when *γ* = 0, only recipient’s features are considered for categorization, and when *γ* = 0.5, both agent and recipient are considered equally.

In total, we introduce three global parameters: a concentration parameter *α* > 0, a Dirichlet prior *β* ≥ 0, and a focus parameter *γ* ∈ [0,1]. Dirichlet prior *β* and focus parameter *γ* together decide the mean feature vector $\mu ^{(z_{i})}$ for category *z*^(*i*)^. Equations 5–7 provide the full definition for featural similarity-based categorization (left column, Fig. 2).

Take the generalization tasks in Fig. 2 as an example. Assuming we saw a blue-square agent causing a red-diamond to become a blue-diamond, but then we need to make predictions about a red-square agent and a yellow-diamond agent. According to the model, both square objects have a high probability of falling into the same “square agent” category and hence sharing the same causal power. However, a yellow-diamond has no shared feature with a blue-square, hence it is more likely to belong to a different category and have potentially different causal powers than a blue-square.

### Latent Causal Categories

Finally, we combine causal functions and object categories into causal categories. The core assumption is that objects within the same causal category share a same causal function:

8 $$ \begin{array}{@{}rcl@{}} P(z^{(i)}|\mathbf{d}, \mathbf{w}) &{=}&\! P(z^{(i)}|d^{(i)}, \mathbf{w}, z^{(-i)}) \\ &{\propto}&\! P(z^{(i)}|z^{(-i)}) P(a^{(i)}, r^{(i)}|\mathbf{w}_{z^{(i)}}) P(r'^{(i)}| a^{(i)}, r^{(i)}, \mathbf{w}_{z^{(i)}}) \\ &\propto& P(z^{(i)}|z^{(-i)}) P(a^{(i)}, r^{(i)}|\mu^{(z_{i})}) P(d^{(i)}|f^{(z_{i})} ) \end{array} $$

Equation 8 adds a causal function component onto Eq. 5. On the final line of Eq. 8, the three products correspond to Eqs. 6, 7, and 2 separately. In other words, the priors for constructing causal categories are provided by

9 $$ \begin{array}{@{}rcl@{}} z^{(i)}|z^{(-i)} &\sim \text{CRP}(\cdot|\alpha) \\ \mu^{(i)} &\sim \text{Dir}(\cdot|\beta) \\ f^{(z_{i})} &\sim \mathcal{G}(\cdot) \end{array} $$

And likelihoods are given by

10 $$ \begin{array}{@{}rcl@{}} a^{(i)}, r^{(i)}|\mu^{(z_{i})} &\sim \text{Dir}(\ \cdot\ |\mu^{(z_{i})}, \beta) \\ d^{(i)}|f^{(z_{i})} &\sim f^{(z_{i})}(a^{(i)}, r^{(i)}) \end{array} $$

When learning data points are abundant, it is impossible to compute the posterior directly because we do not know how many categories are there in advance. We can approximate the posterior distribution using Gibbs sampling. To achieve this, we construct a chain of samples where for each iteration, we sample a causal category for a random observation *d*^(*i*)^ while fixing the category assignment to the other observations, and a sampled causal category *z*^(*i*)^ will then update the category parameters $\mu ^{(z_{i})}$ and $f^{(z_{i})}$. The category sampling step of this Gibbs sampler follows Eq. 8, and the local parameter update step follows definition of computing these parameters given objects in this category. When the number of iterations $n \to \infty $, the sampled categories $\tilde {Z}_{n}$ converge to the true posterior.

With a posterior over causal categories in place, we can make normative generalization predictions to new cases. Similar to Eq. 4, upon observing a partial data point *d*^∗^ = (*a*^∗^,*r*^∗^,⋅), an optimal decision can be made by aggregating the posterior predictive distribution of each possible *r*^′^^∗^ value:

11 $$ \begin{array}{@{}rcl@{}} &&{}P(\tilde{d^{*}}) \propto {\int}_{z} p(\tilde{d^{*}}|z)P(z|d)\text{d}z \\ &&\quad{\approx} \frac{1}{|\tilde{Z}|} \sum\limits_{\tilde{z} \in \tilde{Z}} p(r'^{*}|a^{*}, r^{*}, f^{(\tilde{z})})P(a^{*}, r^{*}|\mu^{(z)})P(z|d) \end{array} $$

and taking the maximum over this predictive posterior

12 $$ \text{Choice} = \arg\max P(\tilde{d^{*}})  $$

Take the two generalization examples in Fig. 2 again. After watching a blue-square agent turning a red-diamond object blue, the posterior distribution over causal functions provides a pool of causal functions these objects may have. For the sake of the example, assume the most salient causal function is that the blue-square object transfers its color to other objects. When making generalization predictions for a red-square object, its shared square feature with the blue-square object leads us to guess they belong to one same category; hence, this red-square object might also transfer its color to other objects. When facing a yellow-diamond object, we are less certain in applying the same causal function. Thus, we are more likely to draw upon the prior distribution of causal laws to account for our uncertainty.

## Experiment 1: One-Shot Generalization

We first investigate the one-shot generalization case: Given a single observation of a causal interaction, will people form consistent causal generalization predictions to new objects? Can we identify the inductive biases behind their generalization choices?

### Method

#### Participants

One-hundred-and-twenty participants (53 female, aged 40 ± 11) were recruited from Amazon Mechanical Turk and were paid $1.19. The task took 5.23 ± 3.17 min. No participant was excluded from analysis. Both Experiments were approved by the Research Ethics panel at the University of Edinburgh.

#### Stimuli and Design

Participants were told that they were making predictions about the behavior of a magic world containing magic stones (agents) and normal stones (recipients). In short videos, participants observed a magic stone collides with a normal stone and appears to alter the normal stone’s color and/or shape (see Fig. 1). Magic stones had a thick border while normal stones had no border. We manipulated two object features—color {*red, yellow, blue*} and shape {*circle, square, diamond*}, leading to 3 × 3 = 9 possible configurations for each object and a nominal 9 × 9 × 9 = 729 configurations of agent and of recipient both pre- and post- the causal interaction.

**Fig. 3:**
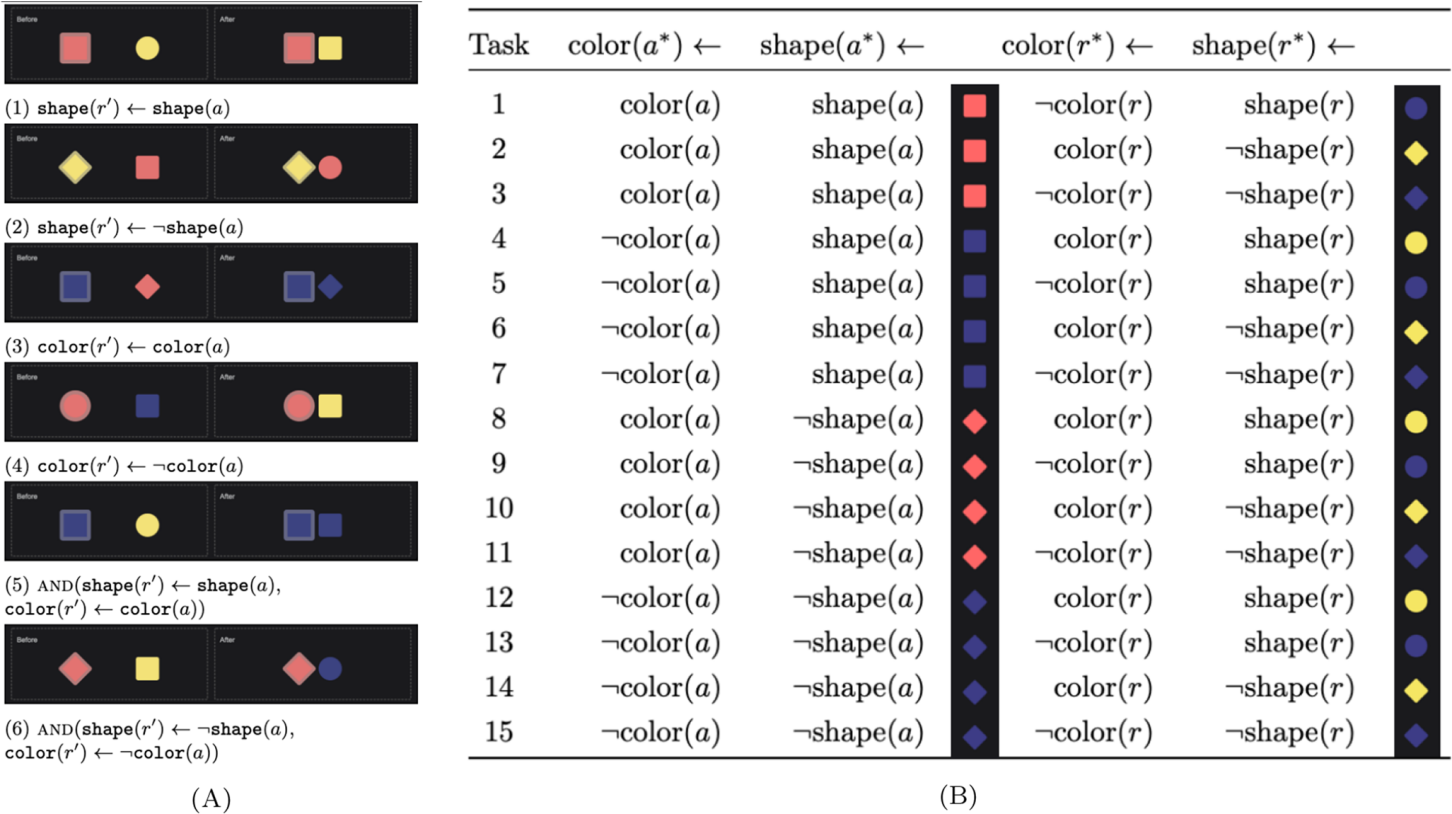
Experiment 1 stimuli. (A) Learning conditions, showing objects before and after a causal interaction. (B) Generalization task configurations *a*^∗^, *r*^∗^ are the agent and recipient in each generalization task; *a* and *r* are the agents and recipients in the learning example. Example stones are for learning condition A1

We used a 6 × 2 between-subject design. There were six learning examples varied between subjects (Fig. 3A)—each participant saw one. Each learning example demonstrates a causal effect differing in whether it results in a change to one or both features of the recipient object, and whether either or both of these new values match the agent object’s features. Note that the function descriptions were not shown to participants and are by no means the only possible way to characterize the causal relationship being displayed.

For each learning example, we constructed 15 generalization tasks by varying object features systematically from the learning example (Fig. 3B). For example, A1 in Fig. 3A depicts a *red square* agent and a *yellow circle* recipient, and according to the specifications in Fig. 3B, generalization task 1 for A1 has a *red square* agent, and a *blue circle* recipient. We call the sequence of tasks from 1 to 15 “near-first transfer” because this sequence of tasks starts with those that differ by only one feature from the learning example and progress to scenes in which all of the features differ. Conversely, we call the sequence of tasks 15 to 1 the “far-first transfer” sequence, because it starts with sets of stones that are completely different from those in the learning examples and progresses back to the more similar cases. Within each sequence, whether the set of different-color tasks or the set of different-shape tasks appeared first (task 1 & 2, 5 & 6, 9 & 10, 13 & 14, 4—7 & 8—11) was shuffled to counterbalance feature order.

#### Procedure

After instructions, participants had to pass a comprehension quiz to start the main task. The main task contained a learning phase and a generalization phase. During learning, participants watched one specific magic stone’s effect on a normal stone (Fig. 1A–C, Fig. 3A), and they could replay the effect as many times as they wanted. After that, participants were asked to make predictions for 15 new pairs of magic stones and normal stones sequentially, by selecting from a panel of 9 possible stones (Fig. 1D). A summary of the learning example (as used in Fig. 3A) was displayed at all times and the animation was replayed once between each generalization task to ensure it was not forgotten. A demo of the task is available at http://bramleylab.ppls.ed.ac.uk/experiments/bnz/magic_stones/index.html.

### Results

We are initially interested in assessing the level of agreement between participants on each generalization task, as this gives a sense of how systematic or strong preferences for any particular patterns of generalization are. We use Cronbach’s alpha (Cronbach, 1943) to measure inter-person consistency. Specifically, the Kuder-Richardson Formula 21 (KR-21)^2^ (Kuder and Richardson, 1937):

13 $$ \rho_{\tau} = \frac{k}{k-1} \left( 1-\frac{kp(1-p)}{{\sigma^{2}_{X}}} \right) $$

where *k* is the number of participants assigned to each condition, *p* is the chance probability of picking an object if responding randomly ($p=\frac {1}{9}=0.11$), and *X* is the vector of aggregated participant selections for each option.

Task-wise consistency *ρ*_*τ*_ demonstrates that participants made systematic one-shot causal generalizations. Across 12 conditions × 15 tasks = 180 tasks, *ρ*_*τ*_ = .80 ± .22. Fisher’s exact test confirmed that participants’ generalization consistency is significantly above random selections, *p* < 0.001. This is therefore another example of human capacity to make systematic one-shot causal generalizations (Kemp et al., 2007).

**Fig. 4:**
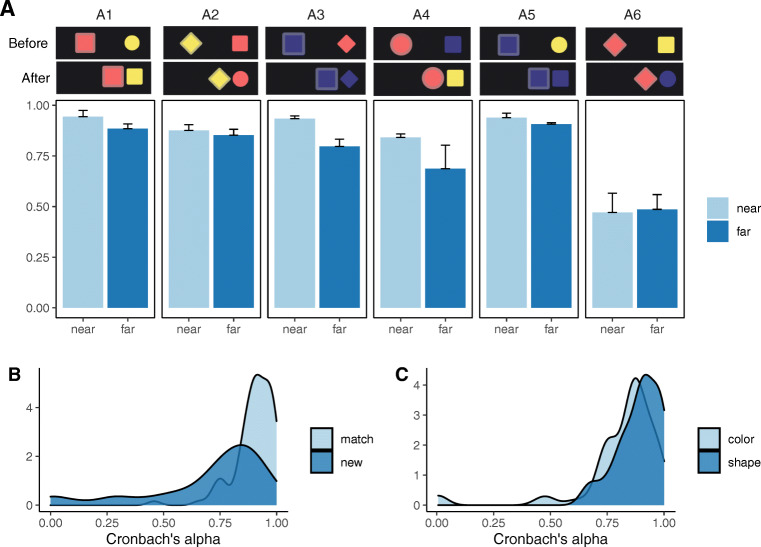
Experiment 1 behavioral results. A: *ρ*_*τ*_ for each learning scene condition (as labeled in Fig. 3A) and sequence order: light blue = *near-first transfer*, dark blue = *far-first transfer*. B: Density distribution of task-wise *ρ*_*τ*_, per match (light blue)/new (dark blue) groups. C: Density distribution of task-wise *ρ*_*τ*_, per color change (light blue)/shape change (dark blue) groups

Next, we compared prediction consistency in the *near-first* and *far-first* transfer order conditions. Generalizations were more consistent overall under near-first transfer: *ρ*_*τ*_ = .83 ± .21, compared with far-first transfer *ρ*_*τ*_ = .77 ± .21, *t*(89) = 3.54,*p* < .001, 95%CI = [0.03, 0.10] (Fig. 4A). *ρ*_*τ*_ was higher for near-first transfer under all learning conditions except A6 “Recipient changes to a new color and shape,” for which both transfer sequences induced low agreement.

Participants also generalized less consistently when the learning task involved new colors or new shapes (Fig. 4B). For learning scenes A1, A3, and A5, where effect states *match* agents’ features, overall consistency was high: *ρ*_*τ*_ = 0.90 ± 0.09. Learning scenes A2, A4, and A6, where effects involved brand *new* values, consistency was lower: *ρ*_*τ*_ = 0.70 ± 0.26, differing significantly from the *match* group, *t*(89) = 6.96,*p* < .001, 95% CI = [0.14, 0.26]. Finally, color and shape changes were generalized to different extents despite these features appearing in symmetric and counterbalanced contexts in the task (Fig. 4C). Shape changes (A1, A2) induced more homogeneous predictions, *ρ*_*τ*_ = 0.89 ± 0.09, compared to color changes (A3, A4) *ρ*_*τ*_ = 0.81 ± 0.19, *t*(59) = 2.88,*p* = 0.005, 95%CI = [0.02, 0.13].

#### Interim Discussion

**Fig. 5:**
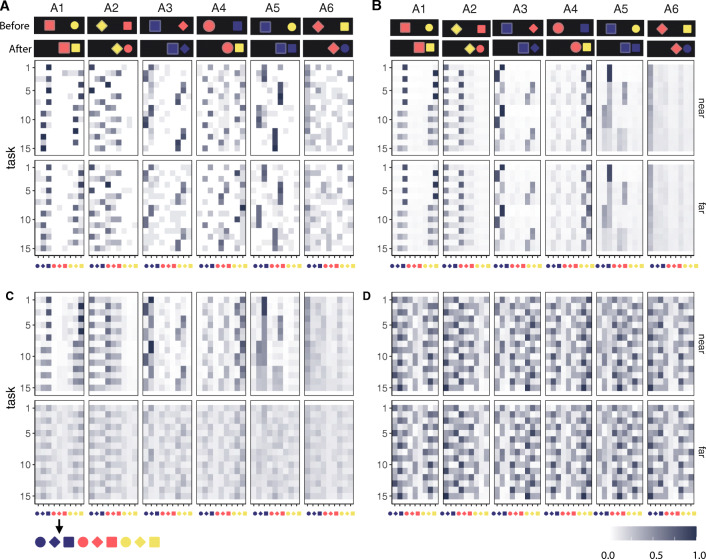
Experiment 1. A. Generalization patterns for all conditions visualized as proportion of participants predicting each stone type for *r*^′^ (column) on each task (row). B. Fitted LoCaLaPro predictions. C–D. Example LoCaLaPro predicted proportions with small *α* (= 0.01) and large *α* (= 8). For both figures, *β* = 0,*γ* = 0.5

We highlight some findings here, and return to them in our “General Discussion”. On the one hand, Experiment 1 demonstrates the strength and consistency of human causal priors, with participants making systematic generalizations from a single example despite these examples being compatible with a very large number of potential causal rules. In Fig. 5A, each cell shows the proportion of participants that selected each object on each task. There is a substantial generalization consistency across participants for most tasks, indicated by the presence of a single dark

On the other hand, we observed a clear departure from normativity providing a clue about cognitive processing, in the form of a generalization order effect. The *near-transfer first* conditions induced more consistent predictions (across subjects), compared to *far-first transfer*. Taking a closer look, for most conditions inter-subject consistency stayed fairly constant across all 15 generalization tasks (Fig. 3B). If there was a high level of agreement about the first generalization—as there tended to be in the *near-first transfer* conditions—participants also tended to make the same predictions as one another right through to the end, even once facing the highly dissimilar scenarios. Conversely, if initial generalizations were diverse (lower homogeneity)—as they tended to be in the *far-first transfer* condition—diversity of judgments persisted until the end, even though the objects in the final tasks were very similar to the learning example. This suggests participants are influenced by their own generalization history in some way.

Generalizations following examples where the recipient is changed to a completely new feature value (A2, A4, A6) induced substantially more diversity in generalization predictions than those that did not (A1, A3, A5). This provides a possible explanation for the particularly low consistency measure *ρ*_*τ*_ in A6. Here, both of the result object’s features are different from those of both the agent and the recipient. Potentially, some participants may have inferred a stochastic rule here such as that agents make recipients take on random feature values. To the extent that participants inferred stochastic rules, we might expect varied predictions even if there is high consistency about the nature of the causal function.

### Models

We now further analyze generalizations in Experiment 1 using our modeling framework. To do this, we fit several model variants to our choice data using maximum likelihood. We then compared them using Bayesian Information Criterion to accommodate for different numbers of parameters.

We first computed a random choice *Baseline*. This model simply predicts $P(\text {choice}=r') = \frac {1}{9}$, for the 9 candidate objects, and has no parameters. We then consider three models based on the modeling framework developed above.

Each model natively provides predictive posterior probability distribution over the nine options, while participants make a single discrete prediction. Thus, for each case, we convert the model’s posterior into discrete choice probabilities using a softmax function to account for decision noise (Luce, 1959). Taking $P_{m}(r'|d)=\{x_{o_{1}}, \ldots , x_{o_{9}}\}$ as the posterior predictive distribution over candidate objects for model *m*, and *t* as an “inverse temperature” parameter:

14 $$ P(\text{choice})= \frac{e^{P_{m}(r'|d)t}}{{\sum}_{x\in r'} e^{P_{m}(r'|d)t}} $$

When *t* → 0, Eq. 14 corresponds to flattening the input distribution toward a uniform distribution while as $t\to \infty $ the input distribution is sharpened, approaching a hard maximization over the probabilities.

#### Universal Causal Laws (UnCaLa)

model uses the causal law induction process to generate a large prior sample of possible causal functions $\tilde {F}$ using the PCFG described earlier (Table 1), then filters this according to the learning example to generate a posterior of potential causal functions consistent with the training data (Eqs. 1–3). It then integrates over these to generate posterior predictions for each generalization task according to Eq. 4. Essentially, UnCaLa assumes that the causal function governing the training case applies universally to all potential generalization scenarios no matter how dissimilar the objects involved may be.

#### Local Causal Laws (LoCaLa)

model captures the idea that multiple latent relationships might be at work, and which will apply to a particular object pair depends on which causal category they fall under. Based on a sample of possible causal functions generated by the PCFG defined in Table 1, the LoCaLa model makes predictions about the result object in a generalization task according to Eqs. 8–11. Note that LoCaLa compares each generalization task with the learning example to make predictions, treating each task as an independent decision problem. Essentially, the more dissimilar a generalization scenario is to the learning example, the less likely LoCaLa thinks it is that the same causal law will apply, meaning it reverts its prediction increasingly toward the prior. How strongly it reverts depends on the concentration parameter *α*: with larger values producing a more drastic return to the prior (Eq. 6). Relatedly, Dirichlet prior *β* captures categorization sensitivity to feature similarity (Eq. 7) with larger values meaning less sensitivity and consequently more noise in the predicted behavioral pattern. Note that we fit *α* and *β*, but fix focus parameter *γ* = 0.5 in Experiment 1, because in this experiment there is no information about what causal categorization assumptions should be preferred. 
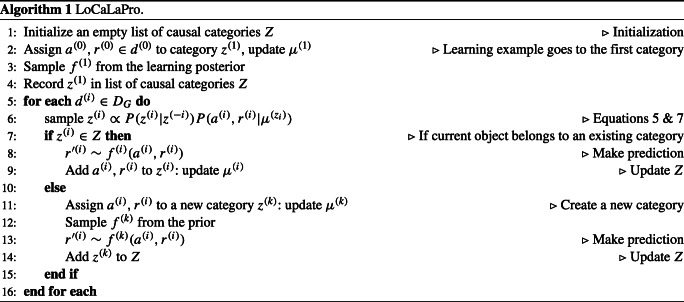


#### Local Causal Laws Process (LoCaLaPro)

model commits to its own causal-category allocations as it makes generalizations, instead of treating each generalization trial independent from each other as in the LoCaLa model. As a result, LoCaLaPro behaves differently when generalizations are made in a different order. To unpack, LoCaLaPro first assigns the object-pair in the learning example to an initial causal category *z*^(1)^ governed by a causal law sampled from the posterior distribution *P*(*f*|*d*). For each generalization task, it then assigns the encountered object pair scenario to either an existing causal category or a new category according to Eq. 5. If an existing causal category is selected, the model simply applies the causal function of this category to make its prediction. If a new category is sampled, however, a new causal law will be assigned to this category. Since there is no evidence about what causal law may apply to this new category, this new causal law is sampled from the prior. Algorithm 1 shows this process in pseudo code.

Instead of approximating a posterior over infinitely many possible categories as the LoCaLa model, the process model LoCaLaPro maintains a small set of available categories that are created online as new generalizations are performed. Furthermore, after categorizing an observation, the LoCaLaPro model updates the list of causal categories *Z* with this categorization decision, reflecting a commitment to its earlier decisions. Concentration parameter *α* thus plays a slightly different role in the LoCaLaPro model as LoCaLa. When *α* → 0, the model becomes increasingly likely to stick with existing categories (Eq. 6). Therefore, under the *near-first transfer* conditions, this model makes predictions closely approximated by the posterior distribution after watching the learning example, throughout the entire generalization phase; in the *far-first transfer* conditions, it is likely to trigger the creation of a new category to accommodate the fact that the generalization scenario drastically differs from the learning example. Subsequent generalization predictions tend to join this newly created category. This induces a generalization-order effect (Fig. 5C). When *α* becomes very large, a new observation has a high probability of being attributed to a new category (Eq. 6), and the overall generalization predictions will simply approach the prior (Fig. 5D). The other hyperparameters *β* and *γ* play the same role as in the LoCaLa model.

#### Model Fits

We used optim function in R to fit the UnCaLa and LoCaLa models to behavioral data. Recall that we generate a large prior sample of possible causal functions $\tilde {F}$ for all three models. In practice, we exploited the fact that each object is composed of two features, and therefore enumerated all the possible causal functions generated by grammar $\mathcal {G}$ up to depth 2. Any causal function in our grammar that is syntactically more complex than those in this set is semantically equivalent to one in this set. With a fixed set of $\tilde {F}$, the UnCaLa model has only one softmax parameter that can be optimized by optim.

LoCaLa has an analytical solution in this case because there is a single learning example, which by definition belongs to category 1. Each generalization task is then compared against the learning example independently. As a result, the chance that a generalization task belongs to category 1 can be computed straightforwardly from parameters *α* and *β*. Assuming the model applies the same *α*,*β* and softmax inverse temperature *t* to each generalization task, we jointly optimize all three parameters to maximize the likelihood of the data using R’s optim function.

For the LoCaLaPro model, since each sampling decision for one generalization task affects how future tasks will be categorized, we can only approximate its posterior distribution with simulation-based method, and optimized parameter values via grid search. Firstly, we set up a coarse grid with *α* = {0.01, 0.1, 0.2, 0.3, 0.4, 0.5, 0.6, 0.7, 0.8, 0.9, 1, 1.5, 2, 4, 8}, *β* = {0, 0.1, 0.2, 0.3, 0.4, 0.5, 0.6, 0.7, 0.8, 0.9, 1, 2, 4, 8, 16, 32, 64, 128, 256, 512, 1024}. For our single shot experiment, in the far-first condition, when *α* = 1, the first generalization observation has a half-half chance to join the learning example or create its own causal category in terms of category size preference (Eq. 6). Therefore, the presence of generalization-order effects in behavioral data indicates that *α* is likely to be smaller than 1. Hence, we included a range of values for 0 < *α* < 1 with fixed-step 0.1, as well as few larger values in case of surprise. For *β*, we take a set of exponentially growing large values up until 1024 in order to accommodate behavioral noise. After running this coarse grid and locating an optimal area, we ran another search over a finer grid for *α* = {0.28,0.30,0.32,0.34,0.36,0.38,0.4,0.42,0.44,0.46,0.48, 0.5,0.52} (*β* is the same as previously) to improve precision.

Table 2 summarizes the model fits. Both the Universal UnCaLa and Local LoCaLa models improve dramatically over the random Baseline, and LoCaLa outperforms UnCaLa in both likelihood and BIC. Figure 5B shows that these computational models indeed predict the dominant judgment patterns among participants. The process model LoCaLaPro best predicts the empirical data. Its fitted *α* parameter for LoCaLaPro is 0.38, confirming the presence of a dominant order effect. The fitted *β* = 1.0, indicating a moderate level of noise in this sample.

**Table 2:** Model fitting results for Experiment 1

	*α*	*β*	*t*	Log likelihood	BIC
Baseline				− 3955	7910
UnCaLa			6.96	− 2761	5529
LoCaLa	2.41	938.81	9.44	− 2748	5518
LoCaLaPro	0.38	1	10.09	− 2736	5494

## Experiment 2: Few-Shot Generalization

Experiment 1 explored one-shot causal generalization, and found evidence of systematic generalization predictions between participants. In Experiment 2, we extend the setup to investigate causal generalization on the basis of multiple complete observations. In addition, recognizing that prediction consistency may not fully imply consistency in causal law induction, we also elicited free guesses about the nature of the causal laws.

### Method

#### Participants

One-hundred-and-sixty-three participants were recruited from Amazon Mechanical Turk. Sixty-one participants were excluded before analysis for failure to provide task-relevant responses.^3^ We thus analyzed 102 participants (37 female, aged 35 ± 10). Each participant was paid $0.50 plus up to $2.30 bonus. The task took 10.4 ± 7.2 min.

#### Stimuli and Design

Similar to Experiment 1, we varied the shape and color properties of the objects. However, instead of using categorical values, we introduced intuitively ordinal feature values. Shapes were all equilateral and differed in terms of their number of sides: 3 (triangle), 4 (square), 5 (pentagon), 6 (hexagon), and 7 (heptagon); colors were of identical hue and saturation (blue) but differed in lightness varying between: 1 (light blue #c9daf8), 2 (medium blue #6d9eeb), 3 (dark blue #1155cc), and 4 (very dark blue #052e54). Staying within the features’ observed values this leads to 4 × 4 = 16 possible configurations for each object, and a nominal 16^3^ = 4096 possible configurations for objects both pre- and post- the causal interaction. These ordinal features enlarge the space of effects and greatly enriches the space of plausible rules, for example allowing causal laws in which a recipient stone becomes *darker* or *lighter* when acted upon, gaining or losing sides, as well as those involving copying or taking specific or random values.

**Fig. 6:**
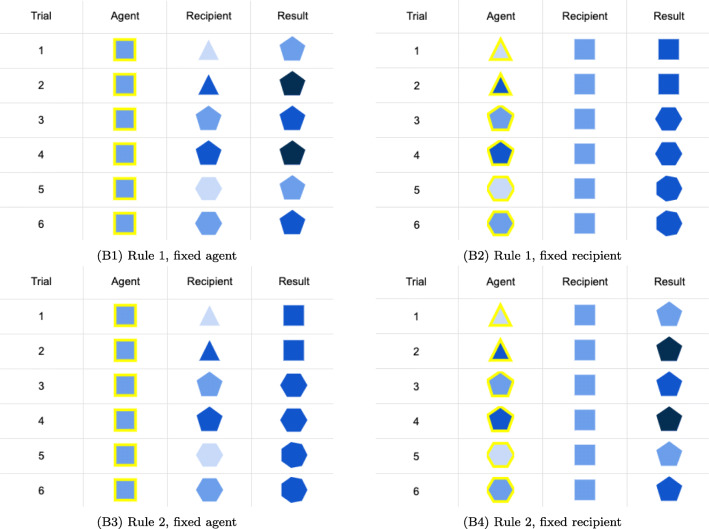
Experiment 2 learning conditions

During learning, each participant observed six causal interactions between different pairs of agent and recipient before making generalizations. We included 2 (evidence-balance) × 2 (ground truth) between-subject factors (see Fig. 6). With respect to evidence-balance, for *fixed-agent* conditions B1 and B3, an identical agent was shown in all learning examples, while the recipients it acted on were varied systematically; in the *fixed-recipient* conditions B2 and B4, the recipient object was always identical but was acted on by six different agents. We designed the evidence to be consistent with two “ground truth” rules that counterbalance between the roles of the shape and the color features:

- **Rule 1** (B1/B2) The recipient gets one increment darker and takes the agent’s shape plus one edge—AND(edge(*r*^′^) ⇐edge(*a*) + 1,shade(*r*^′^) ⇐shade(*r*) + 1)
- **Rule 2** (B3/B4) The recipient gains an edge and takes the agent’s shade plus one shade increment—AND(shade(*r*^′^) ⇐shade(*a*) + 1,edge(*r*^′^) ⇐edge(*r*) + 1)

Note that these “ground truth” rules are just one of an unbounded set of possible universal causal relations consistent with the six learning trials, and a single universal category is just one of a much larger set again of possible local causal law category structures.

We composed generalization tasks according to the configurations in Table 3. In total there were 4 × 4 = 16 generalization tasks for each condition. Additionally, we included two catch-trials for each condition. We randomly chose two learning examples and turned them into generalization trials by hiding the result state. This resulted in 16 + 2 = 18 generalization tasks for each condition.

**Table 3:** Experiment 2 generalization task configurations

	For the fixed object	Instance	For the varied object	Instance
*o*^∗^ =	shade(*o*),edge(*o*)		shade(*o*),¬edge(*o*)	
	¬shade(*o*),edge(*o*)			
	shade(*o*),¬edge(*o*)		¬shade(*o*),edge(*o*)	
	¬shade(*o*),¬edge(*o*)			

*o*^∗^ is the object in generalization tasks, *o* is the object shown during learning. For the varied object, ¬shade(*o*) means picking a shade that has not appeared during the learning phase, and we chose two instances for it.

#### Procedure

After completing instructions, participants had to pass a comprehension quiz to proceed to the main task, consisting of a learning phase, self-report, and a generalization phase. After the main task, participants provided demographic information and feedback. A demo of the task is available at http://bramleylab.ppls.ed.ac.uk/experiments/bnz/myst/p/welcome.html.

Each participant was randomly assigned to one of the four learning conditions (Fig. 6). The six pairs of agent and recipient stones were shown in random order, one after another. By clicking a “Test” button, they could watch the causal interaction as many times as they wanted. After each object pair was tested, a summary visualization of the agent, recipient and the result was added to the top of the page (see Fig. 1E–F)), and remained visible for the rest of the task. After the learning phase, participants were asked to write down their best guess about how the mysterious stones worked, and told they would receive a $0.50 bonus if they described the true underlying causal law. In the generalization phase, participants faced the 18 generalization trials sequentially in random order. For each, participants predicted the result recipient by selecting a number of edges and the shade of blue from two drop-down menus (see Fig. 1F). Participants were instructed they would receive a $0.10 for each correct prediction. We bonused participants as described afterwards.

#### Exclusion Criteria

To check data quality, we screened participants’ self-reports. As with past work, we required workers with Turk approval ratings of above 90%. But in line with Chmielewski and Kucker (2020), we found an unusual number of suspicious responses with very fast completion rates and nonsensical text responses. We thus chose to exclude participants if they failed to provide a task relevant response on the free text guess about the rule. In addition, we checked participant accuracy on the two catch-trials, and found that while overall accuracy is 41%, far above chance (5%), the excluded batch’s accuracy is just 8%, indistinguishable from chance. We provide the full dataset along with the analyzed dataset at https://github.com/bramleyccslab/causal_objects.

### Results

For participants’ generalization predictions, we measured inter-participant consistency as in Experiment 1. To analyze free-text self-reports, we coded them into several categories and ran statistical tests on the coded labels.

#### Generalization Consistency

As with Experiment 1, we measured inter-person consistency in generalization predictions computing *ρ*_*T*_ for the sixteen generalization tasks per condition (excluding the two catch-trials), totalling 4 × 16 = 64 values. Mean consistency was *ρ*_*T*_ = 0.87 ± 0.08, with min *ρ*_*T*_ = 0.57, max *ρ*_*T*_ = 0.98. To compare generalization consistency against random selections, for each condition we conducted Fisher’s exact test on the contingency table of selecting each possible result per trial. For all four conditions, *p* < 0.001. Thus, as in Experiment 1, participants produced systematic generalization patterns.

**Fig. 7:**
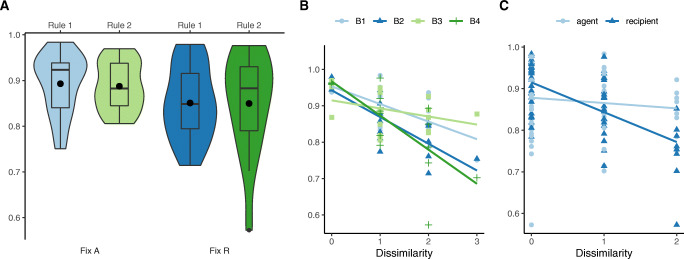
Behavioral results of Experiment 2. All *y*-axes are Cronbach’s alpha values. A: Task-wise inter-person consistency per condition. Violin plots are density. Black dots are mean Cronbach’s alpha values per condition. The major bar in the box plot is the median and the box extent is the 25 and 75 quantiles. B: Inter-person consistency per task differences. C: Inter-person consistency per role differences

We then compared inter-person generalization consistency by condition. As illustrated in Fig. 7A, the *fixed-agent* condition induced higher consistency (*ρ*_*T*_ = 0.89 ± 0.06) than the *fixed-recipient* condition (*ρ*_*T*_ = 0.85 ± 0.1), *t*(31) = 2.12,*p* = 0.04,95*%* CI = [0.001,0.08], while the difference in *ρ*_*T*_ between the ground truth condition was negligible, *t*(31) = 0.22,*p* = n.s. No interaction was detected. In short, participants made more homogeneous predictions after observing the same agent acting on a range of recipients, and diverged more having observed different agents interacting on the same recipient.

Generalization consistency decreased as objects in the generalization tasks become more distinct from those in the learning examples (Fig. 7B). To show this, we constructed a rough measure of *dissimilarity*, by counting the features of generalization trials that took novel values never observed in the learning phase. Formally, let *F*_*L*_ be the set of unique feature values of all the objects appeared during learning, and *F*_*i*_ be the set of unique feature values of objects in a generalization trial *i*, dissimilarity score *DS* = |*F*_*i*_ ∖ *F*_*L*_|. By design, dissimilarity scores *DS* ∈{0,1,2,3} (Table 3). We found a significant negative relationship between task dissimilarity and generalization consistency, *β* = − 0.06,*F*(1,62) = 37.48,*p* < 0.001.

Finally, we fit a linear regression model predicting *ρ*_*T*_ with task dissimilarity, evidence-balance and ground truth, *F*(3,60) = 15.63,*p* < 0.001. This revealed main effects of dissimilarity (*β* = −.06,*p* < 0.001) and evidence-balance (*fixed-recipient*, *β* = − 0.04,*p* = 0.01), but not ground truth (*rule 2*, *β* = − 0.003,*p* = n.s.). As depicted in Fig. 7B, consistency of judgments in the fixed-agent conditions (B1 & B3, lighter lines) decreased slower than the fixed-recipient conditions as dissimilarity increased (B2 & B4, darker lines).

Not only did the evidence-balance condition have a significant effect on generalization consistency, dissimilarity of the agent or recipient objects in the generalization tasks was also associated with lower consistency (Fig. 7C). Holding recipient dissimilarity constant, increasing agent dissimilarity does not predict prediction consistency significantly, *F*(1,62) = 0.77,*p* = n.s.; however, recipient dissimilarity does, *F*(1,62) = 38.8,*p* < 0.001.

#### Self-reports

In Experiment 2, we asked participants to provide an explicit free-text guess about the nature of the causal relationship(s) being tested after they completed the learning phase. Eighty-six percent of these total responses (88/102) were compatible with the relevant learning observations, and here we only analyze these. Two independent coders categorized participants guesses according to their specificity and implicit localization of causal powers. The first coder categorized all free responses, and 15% of the categorized responses were then compared against the second coder’s. Agreement level was 92%. The full set of free responses and the detailed coding scheme are available at https://github.com/bramleyccslab/causal_objects.

Since our ground truths are not the only rules consistent with the learning data, we analyzed participant self-reports not according to whether they got the ground truths right, but whether their own rules were consistent with the learning data, as well as the level of generality in the reports. Hence, we first defined three exclusive and exhaustive response specificity categories: *specific*, *fuzzy*, and *tacit*. A *specific* self-report would predict a unique result object for any potential combination of agent and recipient (for example, “The inactive shape is always changed to a pentagon & its shade is changed to one step darker than the active stone”). Our ground truth rules all belong to the *specific* class of response. A *fuzzy* rule was one that left open for more than one possible result objects (for example, “It will be different colors and shapes”). We distinguished a second form of under-specified self-report, *tacit*, if it left a feature unmentioned, which depending on background assumptions might be taken to imply that feature remained unchanged but could also be compatible with it taking some new or random value (for example, “The active stone adds a side to the inactive stone”).

We also had the coders categorize responses according to whether and how a self-report localized the domain of the causal law asserted. Concretely, we included four labels *A, R, AR*, and *universal*. If a response mentioned a specific context of influence, typically using an *if...* clause, we labeled this according to whether the context mentioned the Agent (e.g., “If the active stone is darker than the inactive stone, it turns the inactive stone darker”), Recipient (e.g., “The active stone causes the other stones to change into a pentagon shape, unless it is already a pentagon shape, in which case it makes it darker”), or both. If a response made no localization or context (e.g., “The active stone cause inactive stones to five sided stone”), then it was labeled as *universal*.

**Fig. 8:**
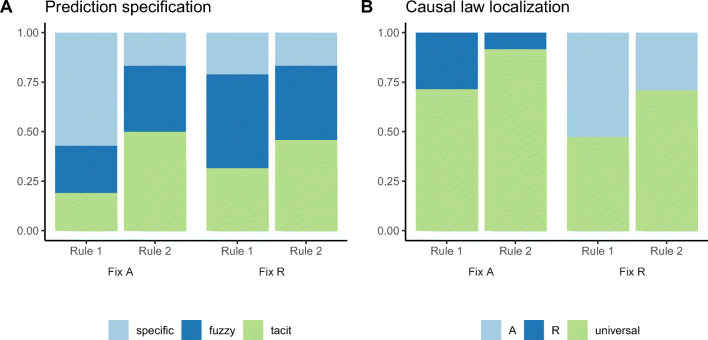
Experiment 2 rule guess categories

Figure 8 illustrates the coding results by learning condition. Guess specificity is summarized in Fig. 8A. We fit a multinomial logistic regression model predicting specificity by evidence-balance and ground truth factors, and found that when taking the *specific* self-report type as baseline, the ground truth factor is a significant predictor for the *tacit* type (*β* = 0.09,*p* = 0.008), while evidence-balance is not. Neither of these two factors is significant for the *fuzzy* type. Figure 8B summarizes participants’ guesses in terms of localization. No participant localized their rule in terms of both agent and recipient. Unsurprisingly, whenever localization occurred, it was applied with respect to the object that varied during the learning phase. A logistic regression predicting universal rule probability by condition showed that both evidence-balance (*fixed-recipient*, *β* = − 1.21,*z* = − 2.3,*p* = 0.02) and ground truth (*rule 2*, *β* = 1.17,*z* = 2.3,*p* = 0.02) were associated with more universal rules. There was no evidence for an interaction, *z* = − 0.5,*p* = n.s.

#### Interim Discussion

These results reveal an asymmetry in causal generalization. In Experiment 2, the two underlying causal relationships induced feature changes that in fact depended critically on both the agent and the recipient. However, participants’ responses suggested they more readily identified the causal effect with the agent object. Consistency was higher for the fixed-agent condition where learners saw the same agent acting on various recipients (B1, B3) than conditions where agent was varied and the recipient was constant (B2, B4). Generalization consistency decayed more slowly when agents became more dissimilar to the training cases than for the matched degree of dissimilarity in terms of the recipient. Self-reported causal laws showed a much higher share of universal causal laws in the conditions where the agent was fixed, and more localization of causal laws was posited when the agents were varied. In fact, causal asymmetry is a well-known inductive bias in physical causation (White, 2006): people tend to judge the “cause” object to be more responsible for bringing the effect even when both objects play equally critical roles. For example, we more naturally think of a moving billiard ball as causing a previously static one to move rather than the static ball causing the moving one to slow down or stop even though the interaction is mathematically symmetric and jointly determined. Experiment 2 thus supports the idea that there is a fundamental causal asymmetry to our causal generalizations.

We also noted a difference between color and shape features in participants’ self-reports. *Tacit* rule guesses were more common for ground truth rule 2. Taking a closer look at *tacit* guesses, we found 91 percent of them (29/32) described only the edge property—the shape feature, and left color changes unmentioned. In Experiment 1, we observed that shape-related changes induced more homogeneous predictions. These findings echo those in the developmental literature suggesting shape is perceived as a more fundamental or “essential” feature (Landau et al., 1988), and therefore more likely to be critical for an object’s causal powers.

### Modeling Results

As with Experiment 1, we compared participants’ generalizations to a random Baseline model, a Universal Causal Laws (UnCaLa) model and a Local Causal Laws (LoCaLa) model, again using maximum likelihood and BIC to account for different numbers of parameters. Since we randomized the presentation of both evidence and generalization trials between subjects, we do not expect systematic effects of the sort accommodated by our LoCaLaPro, so we focus on comparison between UnCaLa and LoCaLa.

We extended the grammar used in Experiment 1 to cover a larger space of ordered feature relationships. Concretely, we introduced + 1, -1, >, < at the “bind relation” step to accommodate potential assertions about the ordering of feature values used in this experiment. Similarly as in Experiment 1, the UnCaLa model is fitted using the optim function in R with one softmax inverse temperature parameter *t*. However, different from the single-shot setup in Experiment 1, in Experiment 2 our LoCaLa model runs over six learning examples with potentially infinite categorizations. Therefore, we used Gibbs sampling to estimate the predictions under each parameterization, and optimized the parameters with a coarse grid search. On each iteration of the Gibbs sampler, one observation is sampled and compared against the other five observations. According to Eq. 6, when *α* = 5 this observation has a 0.5 chance to create its own category in terms of size preference. This probability grows as *α* increases. Therefore, we centered the support values for *α* around 5, with an exponential increase for larger values, resulting in consideration of *α* ∈{1,2,3,4,5,6,7,8,9,10,16,32,64,128,256}. *β* takes the same range of values as in fitting the models in Experiment 1. For *γ*, values of *γ* = 1,0.5 and 0 are of particular theoretical interest, representing localization based on just the agent, agent and recipient equally, or just the recipient. We also included *γ* = 0.25 and *γ* = 0.75 consistent with a mixed focus biased toward either agent or recipient.

**Fig. 9:**
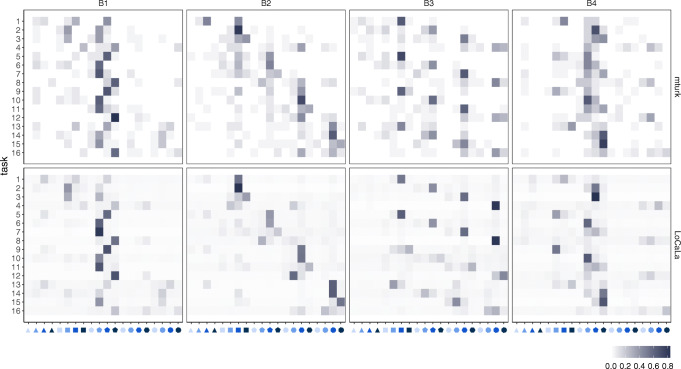
Experiment 2. First row: Generalization patterns for all conditions visualized as proportion of participants predicting each stone type for *r*^′^ (column) on each task (row). Second row: Fitted LoCaLa predictions

We fit UnCaLa and LoCaLa to all 102 × 16 = 1632 data points taken together. Results are summarized in Table 4. Both models improve substantially over the random Baseline, with LoCaLa fitting better than UnCaLa as in Experiment 1. Within LoCaLa, the best fitting *γ* value was 1, indicating that causal categorization was dominated by features of the agents in line with the asymmetric causal attribution bias suggested by our regression analyses. The fitted *α* for LoCaLa is 9 (above chance-level probability of assigning a new causal law to each new observation) confirming the behavioral tendency to create multiple causal categories to account for the evidence. Recall that for the conditions where agent was varied, almost half of the participants reported non-universal causal rules, and when agent was fixed, very few participants’ responses suggested categorization. Here, *γ* = 1 together with *α* = 9 captures this pattern: When observing multiple different agents, participants imputed many local causal laws. When seeing a single agent interact with multiple recipients, they tended to impute a single causal law. The fitted *β* parameter was quite large, as in Experiment 1, this indicates a substantial heterogeneity across participant data taken together. As Fig. 9 shows, our best fitting model indeed visually reproduces participants’ generalization patterns.

**Table 4:** Model fitting results for Experiment 2

	*α*	*β*	*γ*	*t*	Log likelihood	BIC
Baseline					− 4889	9778
UnCala				3.19	− 3706	7417
LoCaLa	9	256	1	9.5	− 3462	6942

## General Discussion

In this paper, we investigated causal generalization based on observations of interactions between objects. Our two experiments demonstrated that people make systematic causal generalizations from one or a few observations and revealed some of the inductive biases that drive these. Participants’ generalization patterns were well-captured by our Bayesian inference model operating on a latent space of causal laws generated by a simple Probabilistic Context Free Grammar prior favoring parsimony, and an extended Dirichlet Process that localized causal laws according to the interacting objects’ features as well as their causal behaviors. Separately, these ideas extend previous work in causal inference and categorization (Bramley et al., 2017; Goodman et al., 2008; Kemp et al., 2010), and in combination they give the first precise formal account of how people (1) partition the world according to causal behavior without relying on innate knowledge—an essential feature of any general model of causal learning (e.g., Griffiths & Tenenbaum 2009; Lucas & Griffiths 2010); and (2) do so in a way that is resource-efficient, requiring modest attention and memory, and supporting snap judgments, albeit at the expense of inducing order effects.

### Beyond Blickets

Our work generalizes the structure of standard “blicket detector” studies, in which different combinations of factors or objects are tested and an effect does or does not occur (e.g., Gopnik et al., 2007; Kemp et al., 2010; Lucas & Griffiths 2010; Sim & Xu 2017), making predictions about a wider family of scenarios while accommodating previous results. If we treat the recipient object’s feature change(s) as a multinomial activation outcome, this can be viewed as analogous to the blicket detector becoming active in the presence of a blicket, and we can use our current framework, unaltered, to see how people reason about a machine’s interactions with prospective individual blickets.

However, our setup puts more emphasis on causal interactions. The collision stimuli we used in our tasks are known to evoke automatic perception of causality (Michotte, 1963), making it an appealing way to study how people reason about cause and effect specifically. In contrast, many studies of causal induction involve descriptions of events that already occurred, or carefully orchestrated demonstrations where combinations of putative causes are presented simultaneously (e.g., Griffiths & Tenenbaum 2009; Johnson & Wk 2015; Steyvers et al., 2003). Such approaches of simultaneously presenting causes are necessary for answering certain scientific questions, but in daily life, we typically observe sequences of changes, which tends to be more informative than an “episodic” approach (Soo & Rottman, 2018). By explicitly distinguishing causal reasoning from a general human ability to discover patterns and learn categories, we are thus able to track inductive biases that are unique to human causal reasoning.

One of those inductive biases we pinpointed is a causal asymmetry. White (2006) argued that people tend to treat the cause object and effect object differently. Our results support this idea: Experiment 2 had pairs of conditions (B1/B2, B3/B4) that shared the same underlying causal relationships, but swapped the dominant presence of the agents and the recipients. If agents and recipients were treated equivalently, this swapping would have had led to symmetric patterns of generalization. In contrast, we observed that participants in conditions B1 and B3 had significantly higher inter-person generalization consistency, and reported inferring fewer, more widely applicable rules. Furthermore, causal asymmetry also presents implicitly in the experiment stimuli: Across our experiments, the agent object stayed the same and only the recipient object went through feature changes. As a result, one may feel that the agent object is more “powerful,” and recipients are “weaker” and susceptible to changes. Future experiments may attempt to disentangle different kinds of asymmetries coming from physical movement or state change.

Last but not least, unlike previous models (e.g., Kemp et al., 2010; Lucas et al., 2014), we are not constrained to binary, present/absent effects, or multiple outcomes, such as different kinds of activations (e.g., Schulz and Sommerville 2006). Our model can also capture higher-order causal relationships, e.g., color/shape matches between blickets and machines (Sim & Xu, 2017). The animations can be extended to investigate more subtle cases such as both agent and recipient objects change features, or agent objects change rather than the recipient object. While we have focused on, and argued for, the advantages of interactions between pairs of objects, our model can also make inferences from simultaneously presented causes by marginalizing over possible orders and intermediate states. Similarly, it can be applied to non-deterministic and conjunctive causes by introducing and marginalizing over hidden features.

### Generalization as Commitment

In Experiment 1 we identified generalization-order effects in one-shot causal generalization. While previous research has shown effects of the order in which learning examples are presented (Danks & Schwartz, 2006; Lu et al., 2016), ours is the first study to find effects of the order in which generalization predictions are made. Many order effects can be understood as a consequence of cognitive agents with limited resources updating their inferences sequentially, for example, anchoring-and-adjustment (Lieder et al., 2012), local updating (Bramley et al., 2017), or amortized computations (Dasgupta et al., 2020). These and other models predict an order effect of *evidence*—people update their beliefs sequentially when there are examples or data to update with. However, our participants made judgments without receiving feedback; our experiments did not vary the order of evidence, so there is no basis for expecting order effects under these previous models.

Our LoCaLaPro model assumes that people implicitly commit to their generalizations as they make them, essentially treating their earlier generalizations as evidence that must be accommodated going forward rather than uninformative guesses that may or may not line up with the ground truth. Consequently, the order in which judgments are solicited can lead to systematic changes in people’s inferences, even in the absence of new evidence. This model predicts the order effects we observed, chiefly that generalization consistency differed between the *near-first transfer* and *far-first transfer* conditions.

### Essentialism

In object cognition, it is well established that shapes and colors are perceived differently (Treisman & Gelade, 1980; Landau et al., 1988), and shape is thought to be taken to be a more fundamental feature than color (Wilcox, 1999). Our behavioral data demonstrate this pattern in a causal setting: In Experiment 1, participants made more systematic generalizations given shape-related effects than color-related effects, indicating that causal laws that are believed to induce more fundamental changes were generalized more consistently; in Experiment 2, participants were predominantly more likely to describe shape-related changes and leave color changes unmentioned, and this tendency prevails the agent/recipient evidence-balance control.

### Causal Representations

While our experiment interface is designed to probe inductive inferences under a strong causal perception, causal representations are also natively built-in to our modeling framework. Our PCFG generates causal functions that explicitly describe the consequence of causal interaction on the recipient object’s features. We allow these to take absolute feature values, like color(r')⇐blue, but also values relative to the agent or recipient’s pre-interaction features, such as color(r') ⇐color(a) or edge(r') ⇐edge(r)+ 1. These causal functions natively capture many kinds of causal theories people may entertain, as confirmed by their self-reports and our model fits (see also Bramley et al., 2018; Goodman et al., 2008; Lake & Piantadosi 2020). Moreover, by grounding causal functions in such object-based representations, these causal functions naturally generalize to novel objects.

Besides being inherently causal and efficiently generalizable, these causal functions are also compatible with the flexibility of human causal reasoning. As Mayrhofer and Waldmann (2015) pointed out, agent-recipient roles and cause-effect roles are separate concepts. Even though agents are usually taken to be the cause, in some cases the static, passive recipients (patients) are actually seen as more causal of an outcome, for instance, a red traffic light being the cause for an active pedestrian to stop walking. Our symbolic grammar makes no assumption about whether agent or recipient object features determine the result, rather, it treats agent and recipient objects equally in its grammar generation process because of its uniform prior (Table 1, row “Relative reference”). In the categorization process, we introduced the focus parameter *γ* that interpolates between considering only the agent, or only the recipient as relevant for what causal function applies. *γ* is later fit to empirical data and yields a best-fitting value of 1, corresponding to categorization by agent-only, confirming the hypothesized causal asymmetry. As a result, our framework is applicable for further investigation into the intricate relationship between agent-recipient concepts and cause-effect roles. For instance, one might estimate an inductive bias controlling the balance of agent and recipient roles in the grammar, or modeling *γ* conditional on learning data.

The applicability of the symbolic grammar generator approach goes beyond these particular causal functions applications. PCFGs can be created for many tasks involving symbolic representations, and indeed have been most traditionally used for modeling language processing (e.g., Johnson 1998). For the PCFGs used in this paper, we included a minimal set of primitives that simply cover the features participants were told about in the instructions. However, recent work has also explored question of whether there is an optimal set of primitive domain-specific-languages (Ellis et al., 2021; Piantadosi et al., 2016). Nevertheless, these modeling choices are not the only way to represent human causal cognition. Our modeling framework is open to, and compatible with, many other options. For example, one may choose to extend the symbolic approach to cover the categorization process as well, or incorporate causal Bayes nets as a representation for causal functions among multiple relata (Griffiths & Tenenbaum 2009; Kemp et al., 2010; Lucas & Griffiths 2010; Pearl 2000, 2009).

### Constructive Cognition

Our modeling framework lines up nicely with a range of recent symbolic accounts of inductive and creative reasoning (e.g., Goodman et al., 2008, 2011; Griffiths & Tenenbaum 2009; Kemp et al., 2010, 2012; Zhao et al., 2018). This framework emphasizes the constructive nature of causal belief formation, in which both the content and extension of our causal concepts are generated rather than pre-specified. The constructive nature of the PCFG calls upon a potentially infinite set of possible causal functions, yet is governed by the preference for parsimony, and encourages systematic composition (see also Bramley et al., 2018; Goodman et al., 2008). The extended Dirichlet Process for category construction goes beyond a hierarchical Baysian modeling approach where categories are pre-defined as inductive biases (e.g., Goodman et al., 2011; Griffiths & Tenenbaum 2009), and thus better captures the flexibility of human generalization behaviors (see also Kemp et al., 2010). This constructive computational modeling framework balances between learning a single causal law versus making generalization predictions based on multiple causal categories, and with the “creating new categories only when on demand” assumption for a process account, our model successfully reproduces the generalization-order effects in behavioral data.

This constructive view of cognition is not unique to causal cognition. Generative grammars have been proven useful in many other fields such as concept learning and category induction (Lake et al., 2015; Goodman et al., 2008; Piantadosi et al., 2016). Symbolic approaches enable compositionality and systematicity, while the sub-symbolic techniques, especially the fast, incremental approximations, make this more scalable to real-world data (Bramley et al., 2017). This framework also draws a close link with probabilistic program induction models (e.g., Bramley et al., 2018; Ellis et al., 2021; Lake et al., 2015; Lake & Piantadosi 2020), where causal beliefs and concepts can be viewed as programs, and accurate generalizations can be viewed as a evidence for successful program synthesis whereby these programs increasingly reflect the true causal laws of nature. We believe our modeling framework can be extended to broader generalization cases beyond causal cognition, and contributes to the collective effort for a hybrid approach in understanding human cognition (Lake et al., 2017; Oaksford et al., 2007; Valentin et al., 2021).

## Conclusion

Across two experiments, we studied how people generalize causal laws from observations of interactions between objects. Participants made systematic causal generalizations after one (Experiment 1) or several (Experiment 2) observations despite there being a large number of potentially compatible explanations. We could explain this pattern with an inductive bias toward simplicity embodied by a hypothesis generation process that favors simple rules and few categories. In addition, we found an intriguing generalization-order effect, and could account for it by treating one’s own earlier judgments as evidence when making new generalizations. We also found evidence for a causal asymmetry in Experiment 2, that we could capture with a model that preferentially localizes causal laws based on the properties of agents. We expect further experiments to unlock the full potential of the proposed computational modeling framework.

## Footnotes

1 Throughout this paper, we use the term *recipient* as equivalent “patient” in the causal learning literature, e.g., Mayrhofer and Waldmann (2015).

2 KR-21 is a simplified version of Cronbach’s alpha known to be more conservative. The resulting consistency measure *ρ*_*τ*_ ranges between 0—indicating uniform spread across all selections—and 1—indicating perfect agreement between participants.

3 Data for Experiment 2 was collected summer 2020 at the height of the COVID-19 pandemic. See the “Exclusion Criteria” section for data quality control.
